# Synthesis of the Marine Bromotyrosine Psammaplin F and Crystal Structure of a Psammaplin A Analogue 

**DOI:** 10.3390/molecules15128784

**Published:** 2010-12-02

**Authors:** Qianjiao Yang, Dan Liu, Deyang Sun, Sen Yang, Guodong Hu, Zuti Wu, Linxiang Zhao

**Affiliations:** Key Laboratory of Structure-Based Drug Design & Discovery of Ministry of Education, Shenyang Pharmaceutical University, Shenyang 110016, China

**Keywords:** psammaplin F, marine bromotyrosine, Cleland’s reagent, total synthesis, X-ray crystal structure

## Abstract

Psammaplin F, an unsymmetrical disulfide bromotyrosine, was isolated from the sponge *Pseudoceratina purpurea* in 2003. We reported here the first total synthesis of psammaplin F in 12% overall yield by employing Cleland’s reagent reduction as key step. The longest linear synthetic sequence starting from 3-bromo-4-hydroxybenzaldehyde and hydantoin was seven steps. In addition, a detailed X-ray crystal structure analysis of psammaplin A analogue **8b** is given for the first time.

## 1. Introduction

The psammaplin disulfide bromotyrosine derivatives exhibit wide-ranging biological activities including anticancer activity [1,2], anti methicillin-resistant *Staphylococcus aureus* (MRSA) activity [3], antifungal activity [4] and antiangiogenic activity [5]. As a result, these natural products have become interesting targets for synthesis on account of their activities, but only a few successful synthetic routes have been reported [3,6,7] over the years. Psammaplin F (**1**) which has been isolated from the sponge *Pseudoceratina purpurea*, containing an oximic amide unit and an oxalamic amide moiety rarely found in marine organisms, is a potent histone deacetylase (HDAC) inhibitor (IC_50_ = 2.1 ± 0.4 nM) [1]. In addition, the combination of a medium chain fatty acid or salt having a carbon chain length of 6 to 20 carbon atoms and psammaplin F (**1**) in oral dosage form can promote absorption of psammaplin F (**1**) in the gastrointestinal tract cell lining [8]. Based on this consideration, and as a continuation of our chemical and biological investigations of marine bromotyrosine natural products, our interest was focused on the synthesis of psammaplin F (**1**), and in this paper the first total synthesis of compound **1** is presented.

## 2. Results and Discussion

### 2.1. Chemistry

**Figure 1:**
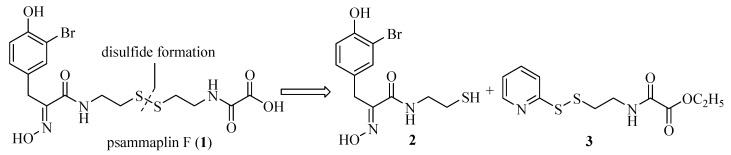
Retrosynthetic analysis of psammaplin F (**1**).

Our retrosynthetic analysis of psammaplin F (**1**) is shown in Figure 1. It was envisioned that the natural product could be synthesized by concise unsymmetrical disulfide coupling [13] between the left hand oximic amide **2** and the right side oxalamic amide **3**, followed by the hydrolysis of the ester. 

**Scheme 1:**
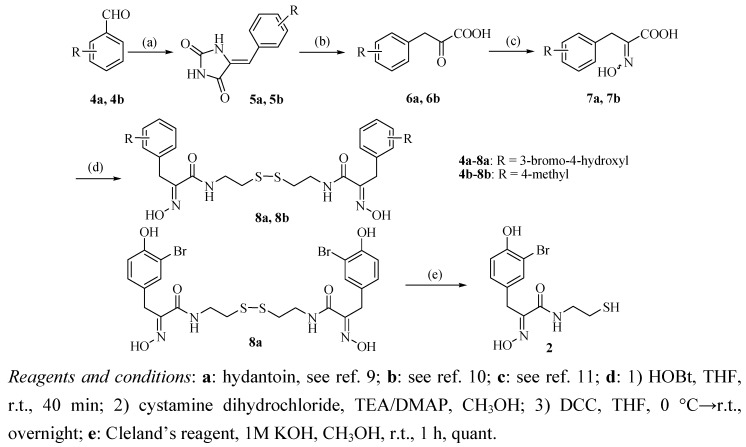
Synthetic route to the target compound **2**.

The synthetic route to oximic amide **2** is seen in Scheme 1. The synthesis started from commercially available 3-bromo-4-hydroxybenzaldehyde (**4a**) and hydantoin, whose Claisen-Schmidt reaction gave the corresponding benzalhydantoin **5a**. 

After hydrolysis and oximation of key intermediate **5a**, the resulting oximic acid **7a** was coupled with cystamine dihydrochloride (**9**) in a one-pot reaction to give psammaplin A (**8a**) in 37% yield for the four steps. 

The co-crystallization of psammaplin A (**8a**) with chitinase B could not fully confirm the skeletal configuration of psammaplin A due to its flexibility [4], therefore, psammaplin A analogues with different substituents in the phenyl ring were also synthesized by this procedure and single crystals of these compounds were grown in order to confirm the configuration of the skeleton. As a result, starting from 4-methylbenzaldehyde (**4b**) and hydantoin, psammaplin A analogue **8b** was obtained in 44% yield for the four steps, a crystal of compound **8b **was grown and the structure confirmed by X-ray crystal structure analysis, which showed that the oxime of these compounds has the (*E*)-configuration. In a word, the structure of synthetic psammaplin A (**8a**) was in accordance with natural psammaplin A reported by Piña *et al*. Psammaplin A (**8a**) was then reduced by Cleland’s reagent to afford oximic amide (**2**) in quantitative yield [14]. 

**Scheme 2:**
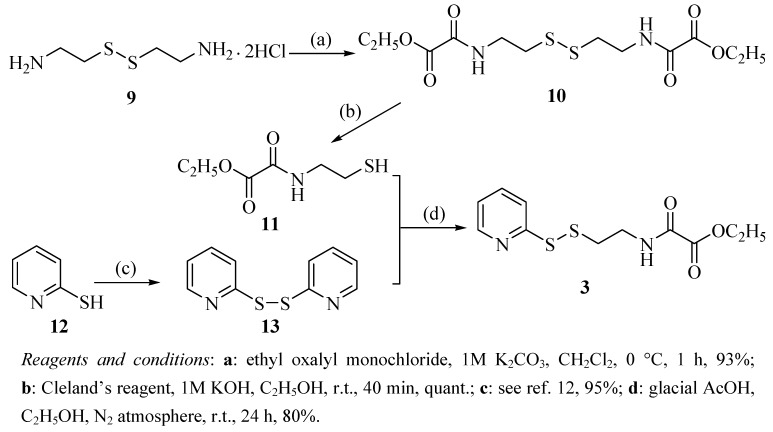
Synthetic route to the target compound **3**.

The synthesis of oxalamic amide **3** is outlined in Scheme 2. Cystamine dihydrochloride (**9**) was converted to bisamide **10** according to a Schotten-Baumann-like reaction followed by reduction to yield oxalamic acetate **11**, which was reacted with aldrithiol (**13**) obtained from the oxidation of 2-pyridinethiol (**12**) to provide the desired oxalamic amide **3** [15]. 

**Scheme 3:**
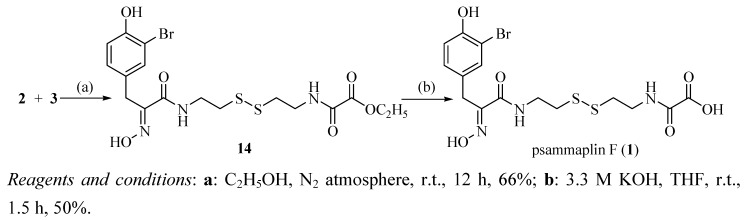
Synthetic route to psammaplin F (**1**).

The convergent synthesis between oximic amide **2 **and oxalamic amide **3 **which is shown in Scheme 3 was achieved based on very simple and mild conditions. The obtained unsymmetrical disulfide **14** was then hydrolyzed by potassium hydroxide in THF-water mixture to give psammaplin F (**1**) [16]. By comparison of ^1^H-NMR and ^13^C-NMR data of synthetic and natural compounds as displayed in Table 1, we were able to prove that the synthetically material was identical to natural psammaplin F (**1**). 

**Table 1:** Comparison of ^1^H-NMR (600 MHz) and ^13^C-NMR (150 MHz) data in CD_3_OD of synthetic and natural psammalin F (**1**).

Position	Synthetic psammaplin F		Natural psammaplin F[1]	
	H (*δ*)	C (*δ*, mult, *J* in Hz)	H (*δ*)	C (*δ*, mult, *J* in Hz)
2	2.85 (t, 6.0)	38.7	2.84 (t, 6.0)	38.6
2’	2.85 (t, 6.0)	38.2	2.84 (t, 6.0)	37.9
3	3.55 (t, 6.0)	39.8	3.54 (t, 6.0)	39.8
3’	3.55 (t, 6.0)	40.0	3.54 (t, 6.0)	40.1
5		166.0		166.0
5’		161.8		161.9
6		153.2		153.3
6’		159.0		159.2
7	3.80 (s)	28.7	3.78 (s)	28.8
8		130.6		130.7
9	7.34 (d, 1.8)	134.5	7.35 (d, 2.0)	134.6
10		110.5		110.6
11		153.7		153.9
12	6.77(d, 8.4)	117.0	6.75 (d, 8.0)	117.2
13	7.07 (dd, 1.8, 8.4)	130.4	7.05 (dd, 2.0, 8.0)	130.5

### 2.2. Crystal structure analysis of compound ***8b***

**Figure 2:**
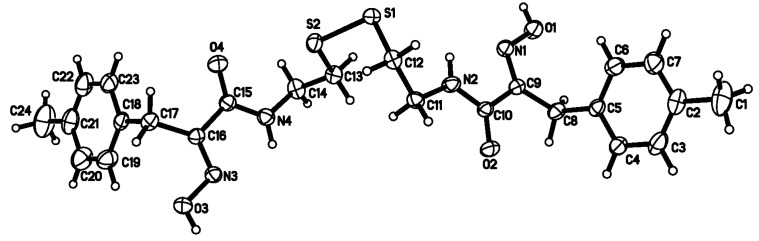
The molecular structure of the compound **8b** with 30% probability of thermal ellipsoids.

The molecular structure of compound **8b **is shown in Figure 2. The detailed crystallographic data, selected bond lengths and angles for compound **8b** are listed in Table 2 and Table 3, respectively. 

**Table 2:** Crystal data and structure refinement for compound **8b**.

Formula	C_24_ H_30_ N_4_ O_4_ S_2_
Fw	502.64
Temp (K)	295(2)
Cryst system	Triclinic
Space group	*P*-1
*a* (Å)	5.7159(9)
*b* (Å)	9.6170(15)
*c (*Å)	23.585(4)
*α* (°)	94.030(2)
*β* (°)	95.455(2)
*γ* (°)	102.532(2)
*V* (Å^3^)	1254.3(3)
*Z*	2
*ρ*_calc _(g cm^-3^)	1.331
*Μ* (mm^-1^)	0.250
F(000)	532
Limiting indices	-6<=h<=6, -11<=k<=5, -28<=l<=27
Data / restraints / parameters	4589 / 0 / 311
θ range for data collection (°)	1.74 to 25.66
Completeness to theta = 25.66	97.2 %
Reflnscollected/unique	6718 / 4589
GOF	1.049
Final R indices [*I*>2*σ*(*I*)]	R_1_^a^ = 0.0484, ωR_2_^b^ = 0.1271
*R* indices (all data)	R_1_ = 0.0595, ωR_2_ = 0.1364

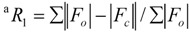
 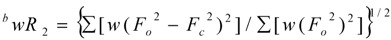

**Table 3:** Selected bond lengths (Å) and angles (°) for compound **8b**.

C(1)-C(2)	1.516(4)	C(14)-N(4)	1.446(3)
C(2)-C(3)	1.361(4)	C(15)-O(4)	1.222(3)
C(2)-C(7)	1.380(4)	C(15)-N(4)	1.337(3)
C(3)-C(4)	1.393(4)	C(15)-C(16)	1.500(3)
C(4)-C(5)	1.375(3)	C(16)-N(3)	1.271(3)
C(5)-C(6)	1.379(3)	C(16)-C(17)	1.501(3)
C(5)-C(8)	1.518(3)	C(17)-C(18)	1.510(3)
C(6)-C(7)	1.382(4)	C(18)-C(23)	1.372(3)
C(8)-C(9)	1.507(3)	C(18)-C(19)	1.376(4)
C(9)-N(1)	1.274(3)	C(19)-C(20)	1.387(4)
C(9)-C(10)	1.502(3)	C(20)-C(21)	1.370(5)
C(10)-O(2)	1.223(3)	C(21)-C(22)	1.360(5)
C(10)-N(2)	1.327(3)	C(21)-C(24)	1.518(4)
C(11)-N(2)	1.445(3)	C(22)-C(23)	1.395(4)
C(11)-C(12)	1.509(3)	N(1)-O(1)	1.384(2)
C(12)-S(1)	1.817(2)	N(3)-O(3)	1.388(2)
C(13)-C(14)	1.518(3)	S(1)-S(2)	2.0376(8)
C(13)-S(2)	1.830(2)		
C(3)-C(2)-C(7)	117.0(3)	O(4)-C(15)-C(16)	120.5(2)
C(3)-C(2)-C(1)	121.2(3)	N(4)-C(15)-C(16)	117.6(2)
C(7)-C(2)-C(1)	121.9(3)	N(3)-C(16)-C(15)	114.33(19)
C(2)-C(3)-C(4)	121.9(3)	N(3)-C(16)-C(17)	127.5(2)
C(5)-C(4)-C(3)	120.7(3)	C(15)-C(16)-C(17)	118.11(19)
C(4)-C(5)-C(6)	117.8(2)	C(16)-C(17)-C(18)	112.58(18)
C(4)-C(5)-C(8)	120.6(2)	C(23)-C(18)-C(19)	117.3(2)
C(6)-C(5)-C(8)	121.6(2)	C(23)-C(18)-C(17)	122.0(2)
C(5)-C(6)-C(7)	120.6(3)	C(19)-C(18)-C(17)	120.7(2)
C(2)-C(7)-C(6)	122.0(3)	C(18)-C(19)-C(20)	121.2(3)
C(9)-C(8)-C(5)	112.76(18)	C(21)-C(20)-C(19)	121.4(3)
N(1)-C(9)-C(10)	113.78(18)	C(22)-C(21)-C(20)	117.5(3)
N(1)-C(9)-C(8)	126.7(2)	C(22)-C(21)-C(24)	120.8(3)
C(10)-C(9)-C(8)	119.52(18)	C(20)-C(21)-C(24)	121.7(4)
O(2)-C(10)-N(2)	122.1(2)	C(21)-C(22)-C(23)	121.6(3)
O(2)-C(10)-C(9)	121.86(19)	C(18)-C(23)-C(22)	121.0(3)
N(2)-C(10)-C(9)	116.09(18)	C(9)-N(1)-O(1)	113.18(18)
N(2)-C(11)-C(12)	110.54(18)	C(10)-N(2)-C(11)	124.39(19)
C(11)-C(12)-S(1)	114.69(15)	C(16)-N(3)-O(3)	112.38(18)
C(14)-C(13)-S(2)	110.52(16)	C(15)-N(4)-C(14)	122.4(2)
N(4)-C(14)-C(13)	113.57(19)	C(12)-S(1)-S(2)	105.05(8)
O(4)-C(15)-N(4)	121.9(2)	C(13)-S(2)-S(1)	104.27(8)

The single crystal X-ray diffraction analysis of compound **8b** reveals that it crystallizes in the triclinic crystal *P*-1 space group and the 2,2'-disulfandiyldiethanamino chain links two 3-(4-methylphenyl)-2-oximidopropionyl groups via the amide group C-N bond. In this structure, all bond lengths and bond angles are comparable to those previously reported [17,18]. The bond angles 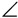 C(9)-C(8)-C(5) and 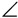 C(16)-C(17)-C(18) are 112.76(18) and 112.58(18)°, respectively. This suggests that the dihedral angles between the amide and oxime group plane and phenyl group are 75.02 and 71.79°, respectively. The two benzene rings are close to parallel, with a dihedral angle of 3.92°. The torsion angles of C(12)-S(1)-S(2)-C(13) and C(11)-C(12)-S(1)-S(2) are -88.50(11) and 77.45(16)°, respectively, which leads to the conclusion that the 2,2'-disulfandiyldiethanamino chain is seriously twisted. It is interesting that the overall shape of the compound **8b** takes on zigzag-like chain. 

**Figure 3:**
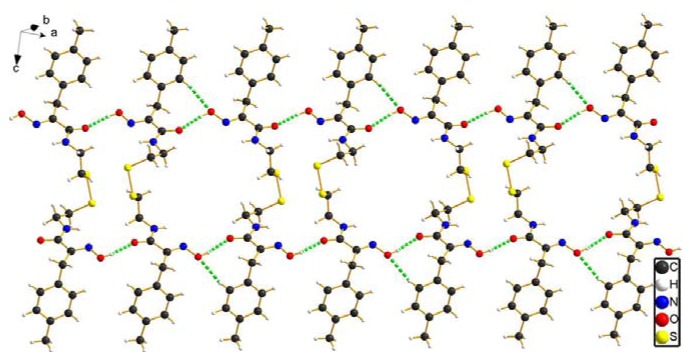
The 1D chain structure of compound **8b** constructed via intermolecular hydrogen bond interactions (green dash line).

Furthermore, intermolecular hydrogen bond interactions [O-H…O] are formed by hydrogen atoms attached to oxygen atoms in the oxime groups and the oxygen atoms associated with carboxyl groups of a neighboring molecule, leading to the formation of ladder-link chains which run along the crystallographic an axis. In addition, intermolecular hydrogen bond interactions [C_sp2_-H…O] between the benzene ring hydrogen atom and the oxygen atom of the oxime group are further favorable to stabilize the ladder-link chains, as depicted in Figure 3. All intermolecular hydrogen bond interaction information is shown in Table 4.

**Table 4:** Intermolecular hydrogen bond lengths (Å) and bond angles (°).

D-H...A	d(D-H)	d(H-A)	d(D-A)	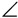 D-H...A
O(1)-H(1)...O(4)#1	0.82	1.87	2.671(2)	167.0
O(3)-H(3A)...O(2)#2	0.82	1.90	2.711(2)	171.5
C(4)-H(4)...O(3)#2	0.93	2.53	3.229(3)	133

Symmetry transformations used to generate equivalent atoms: #1 -x+1, -y+1, -z+1; #2 -x+3, -y+2, -z+1.

## 3. Experimental

### 3.1. General

All melting points (mp) were determined on an electrically heated X4 digital visual melting point apparatus and were uncorrected. IR spectra were recorded on a Bruker IR-27G spectrometer using KBr pellets. NMR spectra were recorded on a Bruker ARX 300 or AV 600 MHz spectrometer at room temperature in DMSO-*d*_6_ or CD_3_OD with tetramethylsilane as an internal standard. Chemical shifts were reported in ppm (*δ*). Mass spectra (MS) were determined on Finnigan MAT/USA spectrometer (LC-MS). High-resolution mass spectra were obtained on Bruker micrOTOF-Q in ESI mode (HR-ESI-MS). Column chromatography was performed with silica gel 60 (200-300 mesh). TLC board (Alugram silica gel G/UV254) was purchased from Macherey-Nagel GmbH & Co. All evaporations were carried out under reduced pressure. All reagents and solvents are commercially available and were used as received.

*General procedure for the synthesis of* *(E,E)-N,N’-bis(3-(substituted* *phenyl)-2-oximidopropionyl)cyst- amines* **8**. To a solution of oximic acid **7** (3.66 mmol) in THF (25 mL) was added HOBt (3.66 mmol) at room temperature. After stirring for 40 min, a mixture of cystamine dihydrochloride (**9**) (1.83 mmol), DMAP (1.10 mmol) and TEA (1 mL) in CH_3_OH (6 mL) were added dropwise at 0 °C, followed by slow dropwise addition of a solution of DCC (4.03 mmol) in THF (25 mL). The resulting mixture was stirred for 1 h and the temperature was then allowed to warm to room temperature overnight. After the reaction was over, the reaction mixture was left standing in a refrigerator overnight and then filtered, the obtained filtrate was washed with brine, dried over MgSO_4_, filtered and concentrated to give **8**. 

*Psammaplin A* (**8a**). The residue was purified by column chromatography (silica gel, petroleum ether-AcOEt 1:1) to give **8a **(0.84 g, 69%) as a white solid, m.p. 170-172 °C (lit. [19] 172-174 °C). IR (KBr): 3384, 2927, 1654, 1626, 1579, 1535, 1493, 1421, 1359, 1284, 1209, 1044, 1015, 984, 801 cm^-1^; ^1^H-NMR (300 MHz, CD_3_OD) *δ*: 2.81 (4H, t, *J* = 6.8 Hz), 3.52 (4H, t, *J* = 6.8 Hz), 3.79 (4H, s), 6.76 (2H, d, *J* = 8.4 Hz), 7.07 (2H, dd, *J* = 8.4 Hz, *J* = 2.1 Hz), 7.36 (2H, d, *J* = 2.1 Hz); ^13^C-NMR (75 MHz, CD_3_OD) *δ*: 28.7, 38.5, 39.5, 110.5, 117.0, 130.4, 130.6, 134.4, 153.1, 153.7, 165.8. LC-MS m/z: 660.9 [M-H]^-^.

*(E,E)-N,N’-bis(3-(4-methylphenyl)-2-oximidopropionyl)cystamine* (**8b**). The residue was purified by column chromatography (silica gel, petroleum ether-AcOEt 2:1) to give **8b** (0.70 g, 76%) as a white solid, m.p. 162-163 °C; IR (KBr): 3392, 3327, 2928, 2852, 1657, 1627, 1576, 1531, 1426, 1204, 1123, 1022, 783 cm^-1^; ^1^H-NMR (300 MHz, DMSO-*d*_6_) *δ*: 2.20 (6H, s), 2.78 (4H, t, *J* = 6.8 Hz), 3.38 (4H, m), 3.73 (4H, s), 7.01 (4H, d, *J* = 8.1 Hz), 7.06 (4H, d, *J* = 8.1 Hz), 8.02 (2H, t, *J* = 5.7 Hz), 11.7 (2H, s); ^13^C-NMR (75 MHz, DMSO-*d*_6_) *δ*: 20.9, 28.8, 37.3, 38.5, 128.9, 129.1, 134.0, 135.3, 152.3, 163.6; LC-MS m/z: 503.3 [M+H]^+^.

*(E)-3-(3-bromo-4-hydroxyphenyl)-2-(hydroxyimino)-N-(2-mercaptoethyl)propanamide* (**2**). To a solution of psammaplin A (**8a**, 0.60 g, 0.90 mmol) in CH_3_OH (30 mL) were added 1M KOH (90 µL) and Cleland’s reagent (0.42 g, 2.70 mmol) at room temperature. The reaction was quenched with 0.5 M HCl at 0 °C after 1 h and extracted with CH_2_Cl_2_. The organic layer was washed with water, brine, dried over MgSO_4_, filtered and concentrated. The desired compound **2**, obtained as a yellow oil (0.60 g, quant.) was used directly in the next reaction. IR (KBr): 3384, 2930, 1655, 1627, 1535, 1493, 1421, 1363, 1285, 1214, 1043, 1009, 801 cm^-1^; ^1^H-NMR (600 MHz, DMSO-*d*_6_) *δ*: 2.24 (1H, t, *J* = 7.5 Hz), 2.54 (2H, q, *J* = 7.5 Hz ), 3.28 (2H, m), 3.69 (2H, s), 6.83 (1H, d, *J* = 8.4 Hz), 7.00 (1H, dd, *J* = 8.4 Hz, *J* = 2.1 Hz), 7.29 (1H, d, *J* = 2.1 Hz), 8.05 (1H, t, *J* = 6.0 Hz), 9.99 (1H, s), 11.8 (1H, s). ^13^C-NMR (150 MHz, DMSO-*d*_6_) *δ*: 27.3, 28.0, 42.4, 109.2, 116.5, 129.1, 129.5, 133.1, 152.2, 152.7, 163.5. LC-MS m/z: 333.0 [M+H]^+^.

*N,N’-bis(ethoxyoxalyl)cystamine* (**10**). To a solution of cystamine dihydrochloride (**9**, 1.00 g, 4.44 mmol) in 1 M K_2_CO_3_ (14 mL) was added dropwise ethyl oxalyl monochloride (1.46 g, 10.6 mmol) in CH_2_Cl_2_ at 0 °C. After stirring for 1 h, the mixture was extracted with CH_2_Cl_2_. The organic layer was washed with water, brine, dried over MgSO_4_, filtered and concentrated. The residue was recrystallized by petroleum ether-acetone 2:1 to give **10 **(1.45 g, 93%) as a white solid, m.p. 102-103 °C; IR (KBr): 3337, 2970, 2932, 1733, 1681, 1535, 1436, 1368, 1309, 1289, 1220, 1058, 1024, 865 cm^-1^; ^1^H-NMR (300 MHz, DMSO-*d*_6_) *δ*: 1.25 (6H, t, *J* = 7.2 Hz), 2.83 (4H, t, *J* = 6.6 Hz), 3.42 (4H, m), 4.22 (4H, q, *J* = 7.2 Hz), 9.01 (2H, t, *J* = 5.7 Hz); ^13^C-NMR (75 MHz, DMSO-*d*_6_) *δ*: 14.1, 36.5, 38.7, 62.3, 157.3, 160.8; LC-MS m/z: 353.1 [M+H]^+^.

*N-ethoxyoxalyl-2-mercaptoethanamine* (**11**). To a solution of bisamide **10 **(0.40 g, 1.14 mmol) in C_2_H_5_OH (30 mL) were added 1M KOH (90 µL) and Cleland’s reagent (0.51 g, 3.42 mmol) at room temperature. The reaction was quenched with 0.5 M HCl at 0 °C after 40 min and extracted with CH_2_Cl_2_. The organic layer was washed with water, brine, dried over MgSO_4_, filtered and concentrated. The desired compound **11**, obtained as a yellow oil (0.40 g, quant.), was used directly in the next reaction. IR (KBr): 3421, 2966, 2927, 1734, 1682, 1528, 1468, 1386, 1292, 1210, 1111, 1014, 858 cm^‑1^. ^1^H-NMR (600 MHz, DMSO-*d*_6_) *δ*: 1.25 (3H, t, *J* = 7.2 Hz), 2.40 (1H, t, *J* = 7.8 Hz), 2.56 (2H, q, *J* = 7.8 Hz), 3.27 (2H, m), 4.21 (2H, q, *J* = 7.2 Hz), 8.99 (1H, t, *J* = 6.0 Hz); ^13^C-NMR (150 MHz, DMSO-*d*_6_) *δ*: 14.3, 23.2, 42.8, 62.5, 157.5, 161.0. LC-MS m/z: 178.0 [M+H]^+^.

*N-ethoxyoxalyl-2-(2-pyridyldithio)ethanamine* (**3**). To a solution of aldrithiol (**13**) (3.01 g, 13.7 mmol) in C_2_H_5_OH (45 mL) were added dropwise glacial AcOH (1.6 mL) and oxalamic acetate **11 **(1.60 g, 4.55 mmol) in C_2_H_5_OH (20 mL) under N_2_ atmosphere. The mixture was stirred for 24 h at room temperature. After evaporation of the solvent, the residue was dissolved in CH_2_Cl_2_ and washed with water, brine, and dried over MgSO_4_. The organic layer was concentrated to afford the crude residue, which was purified by column chromatography (silica gel, petroleum ether-acetone 5:1) to give **3** (1.04 g, 80%) as a white solid, mp 65-66 °C. IR (KBr): 3412, 3214, 2971, 2915, 1729, 1706, 1572, 1538, 1453, 1411, 1369, 1309, 1237, 1182, 1112, 1023, 992, 762 cm^-1^;^ 1^H-NMR (600 MHz, DMSO-*d*_6_) *δ*: 1.25 (3H, t, *J* = 7.2 Hz), 2.96 (2H, t, *J* = 6.6 Hz), 3.44 (2H, m), 4.22 (2H, q, *J* = 7.2 Hz), 7.23 (1H, m), 7.73 (1H, m), 7.80 (1H, m), 8.46 (1H, m), 9.10 (1H, t, *J* = 6.0 Hz); ^13^C-NMR (150 MHz, DMSO-*d*_6_) *δ*: 14.1, 36.9, 38.4, 62.3, 119.8, 121.5, 138.0, 149.9, 157.2, 159.2, 160.7; LC-MS m/z: 287.3 [M+H]^+^.

*((E)-N-3-(3-bromo-4-hydroxyphenyl)-2-oximidopropionyl-N’-ethoxyoxalyl)cystamine* (**14**). To a solution of oxalamic amide **3** (0.26 g, 0.90 mmol) in C_2_H_5_OH (20 mL) was added dropwise a solution of oximic amide **2 **(0.10 g, 0.30 mmol) in C_2_H_5_OH (10 mL) under a N_2_ atmosphere. The whole mixture was stirred for 12 h at room temperature. After removal of the solvent, the residue was dissolved in CH_2_Cl_2_ and washed with water, brine and dried over MgSO_4_. The organic layer was concentrated to afford the crude product, which was purified by column chromatography (silica gel, petroleum ether-acetone 3:1) to give **14** (0.10 g, 66%) as a white solid, m.p. 57-58 °C; IR (KBr): 3378, 2961, 2933, 2873, 1742, 1687, 1658, 1620, 1534, 1416, 1291, 1213, 1115, 1017, 984, 802 cm^-1^;^ 1^H-NMR (300 MHz, DMSO-*d*_6_) *δ*: 1.24 (3H, t, *J* = 7.2 Hz), 2.81 (4H, t, *J* = 6.2 Hz), 3.42 (4H, m), 4.20 (2H, q, *J* = 7.2 Hz), 6.81 (1H, d, *J* = 8.1 Hz), 7.00 (1H, dd, *J* = 8.1 Hz, *J* = 1.8 Hz), 7.27 (1H, d, *J* = 1.8 Hz), 8.06 (1H, t, *J* = 6.0 Hz), 8.10 (1H, t, *J* = 6.0 Hz, 10.0 (1H, s), 11.8 (1H, s); ^13^C-NMR (75 MHz, DMSO-*d*_6_) *δ*: 14.2, 28.0, 36.7, 37.3, 38.5, 38.7, 62.4, 109.2, 116.5, 129.2, 129.5, 133.1, 152.2, 152.7, 157.4, 160.8, 163.6; LC-MS m/z: 508.3 [M+H]^+^; HR-ESI-MS: calcd for C_17_H_22_BrN_3_O_6_S_2_Na^+^ [M+Na]^+^ 530.0026, found 530.0021.

*Psammaplin F* (**1**). To a solution of unsymmetrical disulfide **14** (0.2 g, 0.39 mmol) in THF (8 mL), 3.3 M KOH (1.65 mL) was added dropwise, the mixture was stirred for 1.5 h at 0 °C. The resulting mixture was adjusted to pH 1 with 0.5 M HCl at 0 °C and extracted with AcOEt. The organic layer was washed with water, brine, dried over MgSO_4_, filtered and concentrated. The residue was triturated in Et_2_O, filtered out and dried *in vacuo* to give **1** (0.09 g, 50%) as a white solid, m.p. 122-124 °C; IR (KBr): 3371, 1652, 1535, 1420, 1290, 1209, 1044, 985 cm^-1^; ^1^H-NMR (600 MHz, CD_3_OD) *δ*: 2.85 (4H, t, *J* = 6.0 Hz), 3.55 (4H, t, *J* = 6.0 Hz), 3.80 (2H, s), 6.77 (1H, d, *J* = 8.4 Hz), 7.07 (1H, dd, *J* = 8.4 Hz, *J* = 1.8 Hz), 7.34 (1H, d, *J* = 1.8 Hz); ^13^C-NMR (150 MHz, CD_3_OD) *δ*: 38.2, 38.7, 39.8, 40.0, 110.5, 117.1, 130.4, 130.6, 134.5, 153.2, 153.7, 161.8, 159.0, 166.0; LC-MS m/z: 477.9 [M-H]^-^; HR-ESI-MS: calcd for C_15_H_18_BrN_3_O_6_S_2_Na^+^ 501.9713, found 501.9719.

### 3.2. Single crystal X-ray determination of compound ***8b***

A colorless platelet crystal of the title compound with approximate dimensions 0.36 × 0.25 × 0.08 mm was used for data collection on a Bruker APEX CCD area diffractometer using a graphite-monochromated Mo-*K*α radiation (0.71073 Å) at 295 (2) K in the ω-2θ scan mode. An empirical absorption correction was applied to the data using the *SADABS* program [20]. A total of 6718 reflections were collected in the range of 1.74º < θ < 25.66º, of which 4589 reflections were independent with Rint = 0.0158 and 3743 observed reflections with I > 2σ(I) were used in the succeeding refinements. The structures were solved by direct methods and refined by full-matrix least-squares methods on F^2 ^ using the SHELXTL crystallographic software package [21,22,23]. All non-hydrogen atoms were refined anisotropically. The hydrogen atoms were placed in calculated positions and refined by using a riding mode. Supplementary crystallographic data have been deposited with the Cambridge Crystallographic Data Centre as CCDC No. 786045 (compound **8b**) which contains the supplementary crystallographic data for this paper. The data can be obtained free of charge via www.ccdc.cam.ac.uk/conts/retrieving.html or from the Cambridge Crystallographic Data centre, 12 Union Road, Cambridge CB2 1EZ, UK; fax (+44) 1223-336-033; E-mail: deposit@ccdc.cam.ac.uk.

## 4. Conclusions

In conclusion, we have reported here the first total synthesis of psammaplin F (**1**) from simple starting materials and under mild reaction conditions. The overall yield of psammaplin F (**1**) from 3-bromo-4-hydroxybenzaldehyde (**4**) and hydantoin in seven steps was 12%. In addition, the absolute configuration of (*E*,*E*)-*N*,*N*’-bis(3-(4-methylphenyl)-2-oximidopropionyl)cystamine (**8b**) was confirmed for the first time by X-ray crystal structure analysis.
